# Insight into the Interaction Mechanism of HSA with Aztreonam: A Multispectroscopic and Computational Approach

**DOI:** 10.3390/molecules27227858

**Published:** 2022-11-14

**Authors:** Amal A. Sabour, Altaf Khan, Mohammed R. Alhuzani

**Affiliations:** 1 Department of Botany and Microbiology, College of Science, King Saud University, Riyadh 11451, Saudi Arabia; 2 Department of Pharmacology, Central Laboratory, College of Pharmacy, King Saud University, Riyadh 11451, Saudi Arabia

**Keywords:** HSA, aztreonam, FRET, docking, simulation

## Abstract

Aztreonam is a Gram-negative bacteria-targeting synthetic monobactam antibiotic. Human serum albumin (HSA) plays an important role in the transference of pharmaceuticals, hormones, and fatty acids, along with other compounds, determining their biodistribution and physiological fate. Using several biophysical and in silico approaches, we studied the interaction of aztreonam with HSA under physiological environments in this study. Results confirm the formation of HSA-aztreonam complex where aztreonam showed moderate affinity towards HSA. A static mode of quenching was confirmed from the steady state fluorescence data. FRET findings also showed that there was a significant feasibility of energy transfer between HSA and aztreonam. Site marker displacement experimental conclusion suggested the binding site of aztreonam was the sub-domain IB of HSA. Circular dichroic spectroscopic analysis suggested that aztreonam interaction decreases the α-helical content of HSA. Changes in microenvironment were studied through synchronous fluorescence data. According to molecular docking results, the HSA-aztreonam complex is mostly maintained by non-covalent forces, with a binding energy of 7.7 kcal mol^−1^. The presence of a hydrogen bond, van der Waal interaction, and pi-anion interaction in the binding process, as well as conformational changes in HSA after binding with aztreonam, are all confirmed by molecular dynamic simulation.

## 1. Introduction

Aztreonam is a monobactam antibiotic that is produced synthetically. It works against Gram-negative, aerobic bacteria in the same way that aminoglycosides do. Aztreonam is an antibiotic that has been used to treat a wide range of infections caused by Gram-negative bacteria [1]. However, to assure coverage of anaerobic bacteria as well as Gram-positive, a combination therapy is required in the ministration of mixed infections or diseases of unclear cause. It is administered intravenously, intramuscularly, or via inhalation. When given intramuscularly, however, aztreonam is completely bioavailable. It does not have the nephrotoxic properties that aminoglycosides are known for. It also has a low immunogenicity and has not been linked to coagulation issues [2]. Aztreonam treatment, on the other hand, is frequently associated with increases in liver function tests. Nausea, vomiting, skin rashes, and phlebitis are some of the other side effects linked with its use. Patients allergic to cephalosporins or penicillin tend to be safe when given aztreonam [3].

The solubilization of hydrophobic pharmaceuticals caused by drug binding to plasma proteins aids their distribution to different cells in vivo [4,5]. However, this binding reduces the amount of unbound form of the drug in circulation, which is the pharmacologically active form [6]. In addition, a pharmaceutical with a high affinity for plasma proteins is frequently rendered inert in the biological system [7]. Furthermore, drug binding to a protein may cause conformational changes in protein, which could affect its function. As a result, drug interactions with plasma proteins are thought to be significant in drug pharmacokinetics and pharmacodynamics [8].

The major carrier protein in the circulatory system is human serum albumin (HSA) [9]. It transports endogenous (fatty acids, hormones, amino acids, bile acids etc.) and exogenous (drugs and nutrition) molecules to their target organs via the bloodstream [10]. HSA is comprised of 585 amino acid residues which are mainly composed of helical structure (67%) as evident from crystallographic investigations [11]. The protein is divided into three homologous domains, I, II, and III, which are further classified into two subdomains, A and B [12]. The protein is held together by 17 disulfide bridges [12]. Subdomains IIA and IIIA are the most common site for the binding of drugs and these sites are also known as Sudlow’s Site I and Site II, respectively, [13]. Subdomain IB, in addition to Sudlow’s sites, has been identified as ’as’s third key drug binding region [14]. HSA contains only one tryptophan residue (Trp214) which is located in Sudlow’s site I [15].

Using an array of spectroscopic and computational tools, we conducted a sequence of experiments to determine the binding affinity and mechanism of aztreonam binding with human serum albumin (HSA). UV–vis (UV–visible) absorption and steady state fluorescence spectroscopy were used to assess the energetics of interaction, mechanism of quenching, and complex formation between HSA and aztreonam. Circular dichroism and 3D fluorescence spectroscopy were used to detect the structural and conformational changes after the interaction. The binding site of aztreonam on HSA was also confirmed using site marker displacement studies. FRET analysis was used to unravel the efficiency of energy transfer from HSA to aztreonam. Computational tools were used to decipher the binding site and explore the stability of complex by mimicking the physiological conditions.

## 2. Results and Discussion

### 2.1. UV-Vis Absorption Spectroscopy

**Figure 1:**
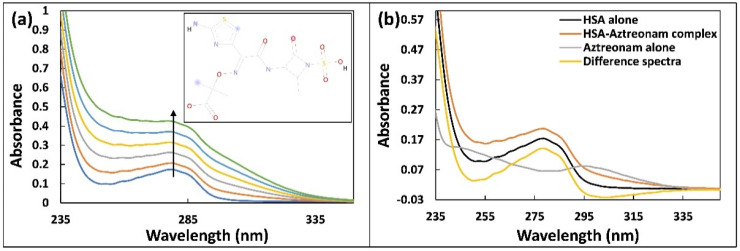
(**a**) UV-visible absorption spectra of HSA (5 µM) titrated with increasing concentration of aztreonam (0–50 µM). The inset of Figure 1a represents the structural formula of aztreonam. (**b**) UV-visible absorption of HSA alone, aztreonam alone, HSA-aztreonam complex, and difference of the spectra of HSA-aztreonam complex and aztreonam alone.

Drug interactions with biological macromolecules such as HSA may cause conformational alterations in proteins that can be studied using spectroscopic tools including UV-vis absorption spectroscopy. The UV-vis spectra in the near region of HSA corresponds to changes in protein tertiary organization, whereas the UV-vis spectra in the far UV region refers to changes in secondary make-up [16]. The UV–vis spectra of HSA (5 µM) alone and complexed with aztreonam (0–50 µM) are shown in Figure 1a to study the changes in the HSA spectra. Gradual aztreonam (0–50 µM) titrations to HSA result in a hyperchromic response that is concentration based. With the addition of aztreonam, a slight blue shift (278 nm to 276 nm) was observed. This shift may be due to changes in polarity around the HSA’s chromophores as a result of aztreonam interaction. The microenvironment around aromatic residues of HSA may also be altered by unwinding of the peptide backbone [17]. To further confirm the formation of complex between aztreonam and HSA, the difference of absorbance spectra of HSA-aztreonam complex and aztreonam alone was calculated as shown in Figure 1b. The difference of the spectra did not overlap with that of HSA alone, confirming the formation of ground state complex between aztreonam and HSA. [18]. This study supported the development of a complex at the ground state between HSA and aztreonam, implying that HSA conformation may change as a result of the interaction with aztreonam.

### 2.2. Steady State Fluorescence

**Figure 2:**
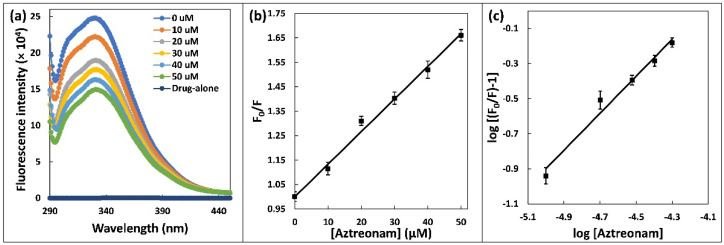
(**a**) Steady state fluorescence spectra of HSA (5 µM) in the presence of increasing concentration of aztreonam (0–50 µM). (**b**) Stern-Volmer plot for the HSA-aztreonam complex. (**c**) Double logarithmic regression plot for HSA-aztreonam binding.

Spectral study based on fluorescence is an effective approach to explore the binding of drugs with proteins [19]. Steady state fluorescence was used to investigate HSA binding with aztreonam. The intrinsic fluorescence signal was recorded before and after the addition of aztreonam at fixed concentration of HSA (5 µM) in the emission range of 290–420 nm. When the concentration of aztreonam was changed (0–50 µM), the fluorescence of HSA decreased, indicating the formation of non-fluorescent complex between HSA and aztreonam, as shown in Figure 2a [20]. Modification in the location of charged groups, a rise in hydrophobicity, or reduction in the surrounding microenvironment polarity can all be attributed for the decrease in fluorescence intensity [18].

To investigate changes in fluorescence intensity at specific wavelengths, the Stern–Volmer equation was used:

(1) $$\frac{F_{0}}{F}=1+K_{\mathrm{sv}}\;\left[Q\right]=1+K_{q\;}\tau_{0}\left[Q\right]$$

The HSA’s fluorescence signals in absence and presence of the quencher are F_0_ and F, respectively [21]. [Q] is the concentration of the aztreonam, which acts as quencher and the Stern-Volmer quenching constant is written as K_sv_. As illustrated in Figure 2b, the K_sv_ value was Stern-Volmer plot which was found to be 1.33 × 10^4^ M^−1^. The Stern-Volmer plot linearity stands for a single class of quenching mechanism, which might be static or dynamic. Using the equation, it was possible to substantiate the nature of binding, either dynamic or static, based on value of K_Q_:

(2) $$K_{Q}=\;K_{\mathrm{sv}}/\tau_{0}$$

where τ_o_ is average lifetime of fluorophore (10^−8^ s). K_sv_ is Stern-Volmer constant and K_Q_ is quenching rate constant [22]. As a result, K_Q_ was calculated to be 1.33 × 10^12^ M^−1^ s^−1^. The obtained value exceeds the limit value necessary for the scatter collision quenching (dynamic) constant (2 × 10^10^ M^−1^ s^−1^). It suggests that quenching process was initiated by the formation of a ground state complex between HSA and aztreonam, rather than by a dynamic mechanism [23]. The number of binding sites (n) and binding constant (K_b_) for the HSA complexed with aztreonam were deliberated using double logarithmic regression equation:

(3) $$\;\log\frac{F_{0-}F}{F}={\mathrm{logK}}_{b\;}+n\;\log\left[Q\right]$$

‘K_b_’ is binding constant, and ‘n’ is number of binding sites. As illustrated in Figure 2c, the intercept and slope of the double log plot yield the values of ‘K_b_’ and ‘n’, respectively. The value of K_b_ was estimated to be 1.109 × 10^4^ M^−1^. As reported, the binding contact for the interaction of aztreonam with bovine serum albumin was found to be 5.68 × 10^3^ M^−1^ at 25 °C [24]. Number of binding site ‘n’ was determined to be 1, confirming the appearance of a single aztreonam binding site in HSA.

### 2.3. Site Marker Displacement Assay

**Figure 3:**
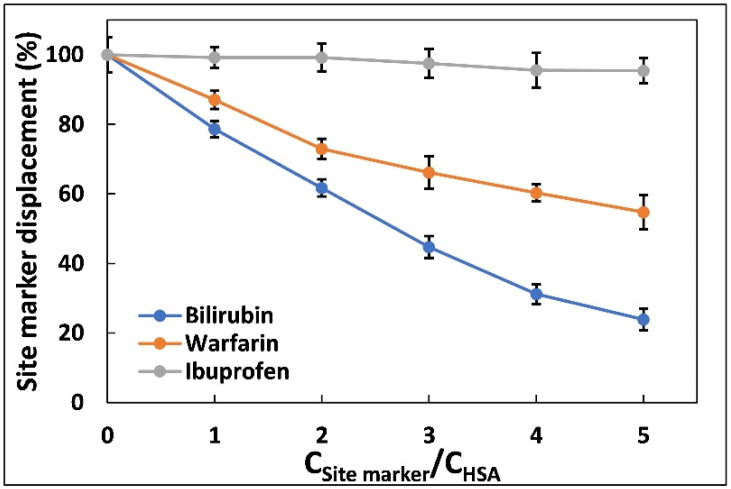
Effect of site probes on the fluorescence emission intensities of the HSA complexed with aztreonam. The experiments were carried out using three site markers (warfarin, ibuprofen, and bilirubin). C_HSA_ = 5 µM, C_Aztreonam_ = 60 µM, C_Bilirubin, ibuprofen, warfarin_ = 0–30 µM), λex = 280 nm, T = 298 K.

Site marker displacement assays were effectuated to further substantiate the binding position of aztreonam on HSA. Three main drug-binding sites are present in HSA structure, essentially named as site I, II, and III. Sudlow’s site I and II are present in hydrophobic cores of subdomains IIA and IIIB [25,26]. Small aromatic carboxylic acid compounds prefer to interact at Sudlow site II, whereas negatively charged and large heterocyclic compounds tend to choose to bind at Sudlow site I. Site III, that supports the binding of polycyclic aromatic hydrocarbon epoxides, is one of the most specialised sites in sub-domain IB [14]. In this experiment, the binding position of aztreonam in HSA were investigated by using warfarin, ibuprofen, and bilirubin. These are well-known site markers for site I, site II, and site III, respectively [27]. Measures of 5 µM of HSA and 60 µM of aztreonam were titrated with the increasing concentrations of warfarin, ibuprofen, and bilirubin. The displacement percentage of aztreonam by the site marker was calculated using following equation:

(4) $$\mathrm{Site}\;\mathrm{marker}\;\mathrm{displacement}\;\left(\%\right)=\frac{F_{2}}{F_{1}}\times 100$$

where F_1_ and F_2_ are the peak fluorescence of HSA-aztreonam complex in the absence and presence of various site markers, respectively. Figure 3 shows a plot of the proportion of site marker displacement values against various molar ratios of site marker to HSA. The proportion of aztreonam displacement from HSA caused by bilirubin was larger than the percentage of aztreonam displacement caused by warfarin and ibuprofen, indicating that the aztreonam binding position, which is located at site-III in subdomain IB of HSA [28].

### 2.4. Synchronous Fluorescence

**Figure 4:**
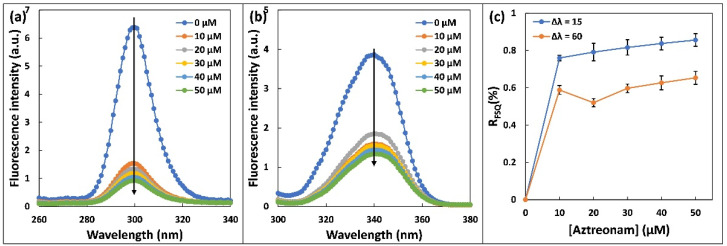
Synchronous fluorescence spectra of 5 µM HSA upon addition of aztreonam (0–50 µM): (**a**) Δλ = 15 nm. (**b**) Δλ = 60 nm, and (**c**) Plot of ratio of synchronous fluorescence quenching (R_SFQ_) versus aztreonam concentration (0–50 µM).

The alterations in microenvironments of Trp and Tyr residues of HSA due to aztreonam interaction were examined by synchronous fluorescence [29]. The excitation and emission monochromators were scanned simultaneously with a constant wavelength interval Δλ (Δλ = λem − λex) between them. The spectrum characteristic of Tyr residues was detected at a wavelength of Δλ = 15 nm, and spectrum characteristic of Trp residues was observed at a wavelength of Δλ = 60 nm [30]. The polarity of their surroundings affects the fluorescence emission peaks of aromatic amino acid residues such as Tyr and Trp. Figure 4 shows the effect of adding aztreonam to the synchronous fluorescence spectra of has at wavelengths of Δλ = 15 and Δλ = 60 nm. The greatest emission wavelengths of Tyr and Trp residues did not turn around much; however, the fluorescence intensity of Tyr declined more than that of Trp. The results suggested that the binding of aztreonam to HSA does not change polarity around Trp and Tyr residues [31]. In addition, the findings revealed that aztreonam binding does not come out to cause significant structural modification in the microenvironment surrounding Tyr residues.

Using the following relationship, the ratio of synchronous fluorescence quenching (R_SFQ_) was also estimated:

(5) $$R_{\mathrm{SFQ}}\left(\%\right)=1-\frac{F}{F_{0}}\times 100$$

The following mathematical relationship was used to compute the synchronous fluorescence quenching ratio (R_SFQ_). In the presence of consecutive additions of aztreonam, F_0_ and F are the synchronised fluorescence intensities of HSA. Figure 4c shows the relationship between R_SFQ_ and aztreonam concentration. It may be deduced that the R_SFQ_ obtained at Δλ = 15 nm is significantly greater than the R_SFQ_ obtained at Δλ = 60 nm, implying that aztreonam binds to HSA around the Tyr residue rather than the Trp residue [32].

### 2.5. Fluorescence Resonance Energy Transfer

The FRET was used to measure the distance between fluorophore of HSA and aztreonam. In this case, Trp of HSA acted as donor and aztreonam acted as acceptor. The efficiency of energy transfer (E), distance of acceptor from donor (r), and critical distance (R_0_), were computed using the equations:

(6) $$E_{\mathrm{Fret}}=1-\frac{F}{F_{0}}=\frac{R_{0}^{6}}{R_{0}^{6\;}+r^{6}}$$

**Figure 5:**
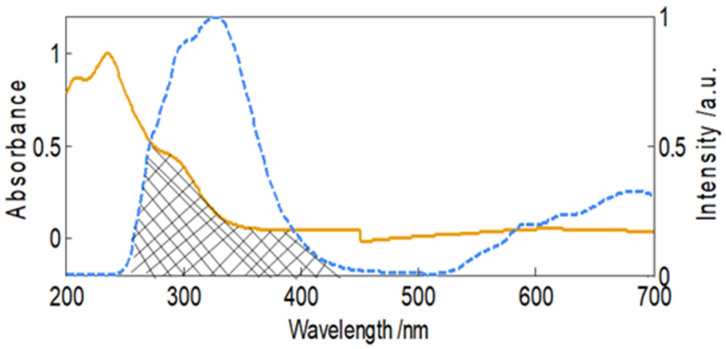
Normalized spectral overlapping of HSA’s fluorescence and aztreonam’s absorbance.

The F_0_ and F are fluorescence of HSA alone and HSA complexed with aztreonam. ‘r’ is distance between HSA and aztreonam, whereas R_0_ is critical distance, and its value may be calculated using the following equation:

(7) $$R_{0}^{6}=8.8\times 10^{-25}k^{2}n^{-4}\varphi J$$

where n represents refractive index and fluorescence quantum yield is denoted by φ. The spectral overlap of HSA’s fluorescence and aztreonam’s absorbance (J) was calculated using the connection below:

(8) $$\;J=\frac{\sum F\left(\lambda \right)\varepsilon \left(\lambda \right)\lambda^{4\;}\Delta \lambda }{\sum F\left(\lambda \right)\Delta \lambda }$$

where F(λ) is fluorescence at wavelength λ, and ε(λ) is molar extinction coefficient aztreonam at wavelength λ. As demonstrated in Figure 5, the absorption spectrum of aztreonam and the fluorescence emission spectra of HSA overlap. The following values E, R_0_, r, and the spectral overlap were obtained as 0.1976, 2.178 nm, 2.75 nm, and 3.4 × 10^12^ M^−1^ cm^−1^ nm^4^, respectively.

### 2.6. Three-Dimensional (3D) Fluorescence

Excitation-emission matrix fluorescence in three dimensions is an inquisitive technique used to investigate the conformational and microenvironmental alterations caused proteins as result of ligand interaction (Qureshi and Javed, 2021). The fluorescence intensity along with maximum emission wavelength are two major parameters which influences the polarity around aromatic amino acids [33]. The microenvironmental changes in HSA after the interaction of aztreonam were investigated 3D fluorescence emission spectral analysis. HSA alone and HSA-aztreonam complex with a molar ratio of 1:6 have different contour maps and 3D fluorescence emission spectra. Table 1 also summarizes the intensity of the characteristic peaks derived from the 3D fluorescence measurements. The 3D fluorescence spectra for HSA alone and HSA-aztreonam adduct revealed four peaks. Rayleigh scattering (λ_ex_ = λ_em_) and second-ordered scattering (λ_em_ = 2λ_ex_) are reflected by peaks ‘a’ and ‘b’, respectively, as previously reported in the literature [34]. The peak ‘1′ is attributed to fluorescence of tyrosine and tryptophan residues. The higher-order excitation of Trp and Tyr residues are observed at peak ‘2′ [35]. It has been determined that following binding with aztreonam, the fluorescence at peak ‘a’ in HSA decreases, implying a decrease in HSA diameter and, as a result, a lowering of the scattering effect. As a result, the complex formation between HSA and aztreonam is also confirmed. In addition, peak ‘1’ and ‘2’ fluorescence signal reduced in the presence of aztreonam, as observed from data, but to varying degrees. As a result of the slight unfolding of the backbone framework of polypeptide chain of HSA, quenching in both peaks was observed with indicates that the binding of aztreonam with HSA causes microenvironmental and conformational changes.

### 2.7. Circular Dichroism

**Figure 6:**
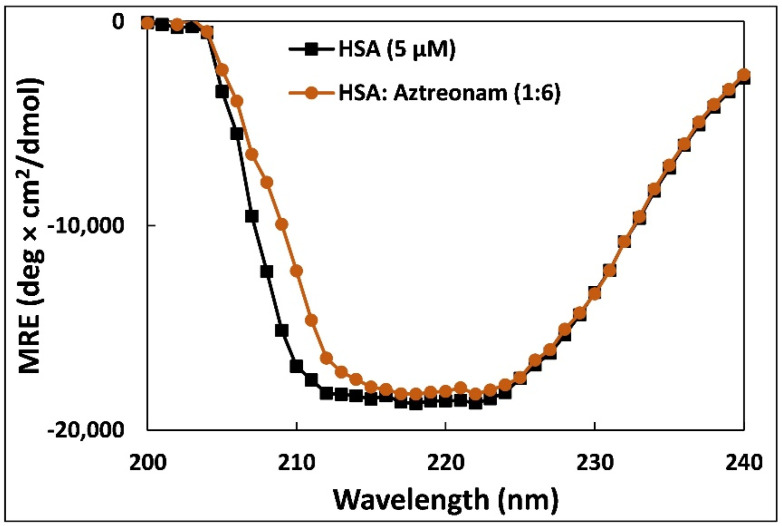
Far-UV-CD spectra of 5 µM HSA in presence of aztreonam added in the molar ratio of 1:6.

Circular dichroism is the preferred approach to monitor the alterations in secondary structures as well as conformational changes when transport proteins bind to small molecules [36]. The changes in the conformation of HSA upon binding to aztreonam was evaluated using CD. Human serum albumin exhibits two characteristic negative bands at 208 and 222 nm designated with an α-helical structure of the HSA [37]. Figure 6 points out clearly the CD spectra of HSA at metabolically active pH in the molar ratios of protein to drug (1:0, 1:6).

The mean residue ellipticity (MRE) is given by following equations [38]:

(9) $$\mathrm{MRE}=\frac{\mathrm{Observed}\;\mathrm{CD}\;\left(\mathrm{mdeg}\right)}{C_{p\;}\mathrm{nl}\;\times 10}$$

where n symbolizes the number of amino acid residues in HSA (585), l implies the cell path length (0.1 cm), and C_p_ is the molar concentration of HSA i.e., 5 µM.

Further, the % α-helical content of HSA solution and HSA complexed with aztreonam (1:6) was calculated using equation at 222 nm [39]:

(10) $$\alpha -\mathrm{helical}\;\mathrm{content}\;\left(\%\right)=\frac{{\mathrm{MRE}}_{222}-2340}{30300}\times 100$$

From the analysis, it was observed that native HSA contain 69% α-helix and HSA-aztreonam complex possess 67%. A slight change in the % α-helical content of HSA after binding with aztreonam suggests that binding leads to secondary structure alteration of HSA. Moreover, the secondary structure was also calculated from BeStSel web server to obtain other secondary structural components [40]. The amount of β-sheets increased by roughly 10% after complexation. Similarly, the turns also increased by more than 5%. However, there was a decrease in other coils by more than 10% following the binding of aztreonam. A study found that no change in α-helical content of HSA was observed after interaction of levofloxacin and sparfloxacin. However, a small change (1.33%) was found after the complexation of enrofloxacin. The change in α-helical content was more pronounced when ciprofloxacin hydrochloride interacted with HSA [41].

### 2.8. Molecular Docking

**Figure 7:**
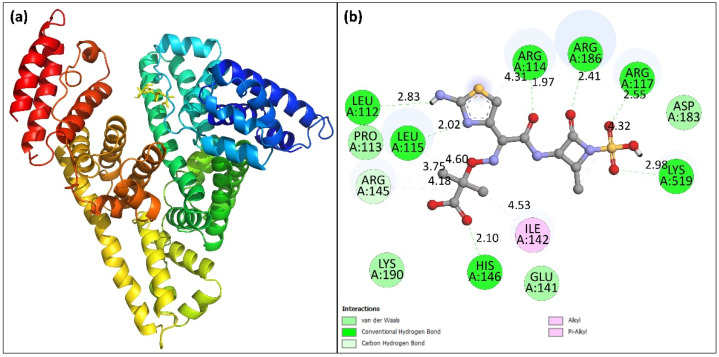
(**a**) Docked pose of aztreonam binding to HSA. (**b**) 2D plot showing the different kinds of force involved in the interaction.

Molecular docking is an in silico binding analysis approach that aids in the discovery of novel medications by elucidating the drug’s binding location to biological macromolecules [42]. This method requires less time and provides more detailed information regarding the interaction between drug and its target molecule in terms of binding energy and affinity [43]. In most cases, the results produced using the in silico method are consistent with those acquired using the in vitro and in vivo methods. To achieve the most energetically stable conformation, the structure of HSA was kept rigid while aztreonam was made flexible. In this study, the docked conformation exhibiting lowest energy was analyzed. Figure 7a shows the docked conformation with the lowest binding energy. The binding energy of best conformation was obtained as −7.7 kcal mol^−1^. The aztreonam binding site in HSA was found in sub-domain IB of HSA at site III, corroborating the site-marker findings. The 2D plot for the aztreonam–HSA binding is shown in Figure 7b. Lys^190^, His^146^, Glu^141^, Ile^142^, Arg^145^, Pro^113^, Leu^115^, Leu^112^, Arg^114^, Arg^186^, Arg^117^, Asp^183^, and Lys^519^ are the amino acid residues located in the proximity of aztreonam that are involved in binding. The 2D plot of HSA interaction docked with aztreonam also reveals that hydrogen bond, van der Waals forces, and pi-anion interactions are all important factors in the binding process. The in silico results confirmed the experimental findings and supported our conclusions.

### 2.9. Molecular Dynamic Simulation

To determine the details of the drug binding process to serum albumin, molecular dynamics (MD) simulations are routinely performed. We selected the lowest energy conformation of aztreonam for further examination using MD simulation simulations based on the docking investigation [44]. It contributed to the establishment of a realistic model of aztreonam binding to HSA in the presence of solvent. Various parameters such as RMSF, RMSD, Rg, SASA, and H-bonding were evaluated from simulated trajectories to assess the stability of the system [45].

**Figure 8:**
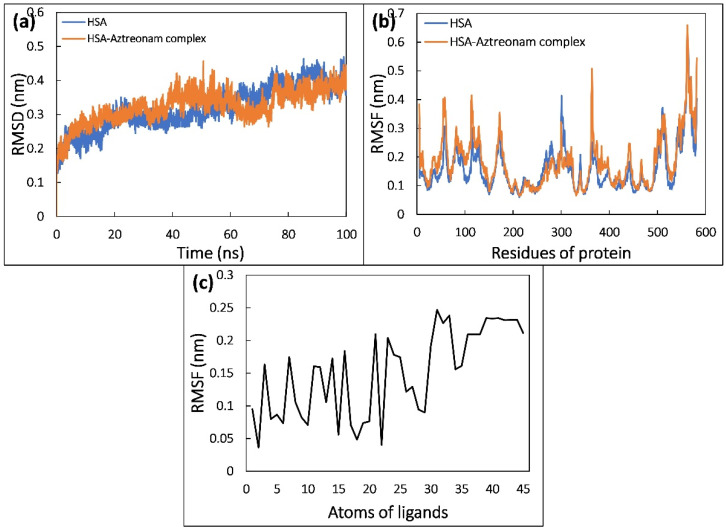
(**a**) Root mean square deviation (RMSD) of backbone of HSA and HSA-aztreonam complex as function of time. (**b**) Root mean square fluctuation (RMSF) of C_α_ atoms of HSA in the absence and presence of aztreonam. (**c**) The average RMSF value of each atom of aztreonam in complexed with HSA.

The RMSD of HSA in the native and complex states was traced and plotted during a 100 ns MD simulation to confirm the binding’s structural stability. As shown in Figure 8a, with reference to the initial structure of HSA and HSA-aztreonam complex, RMSDs were determined. HSA and HSA-aztreonam complexes had an average RMSD of 0.45 nm and 0.44 nm, respectively. The stable interaction of the aztreonam to the binding pocket of HSA is indicated by smaller RMSD values for HSA alone and HSA-aztreonam complex. The RMSD of HSA during simulation reveals that there is just a minor modification in protein structure after aztreonam binding [46].

The RMSF analysis can be used to investigate the average fluctuation of each residue of protein. It can be investigated while simulating the effect of ligand binding to protein. Figure 8b depicts the average RMSF of the HSA and HSA-aztreonam complexes. HSA residues display minor residual fluctuations with and without aztreonam, indicating a minimal change in HSA structure upon aztreonam binding [47].

The average RMSF of every atom of aztreonam was also calculated, and it was revealed that the RMSF of every atom of aztreonam differed significantly from its initial value, indicating a significant shift from its initial position in dynamic manner as shown in Figure 8c. During simulation, a significant dynamic shift of aztreonam contributes to several forms of interactions with neighboring residues, such as shifts in between hydrogen bonds and hydrophobic interactions.

**Figure 9:**
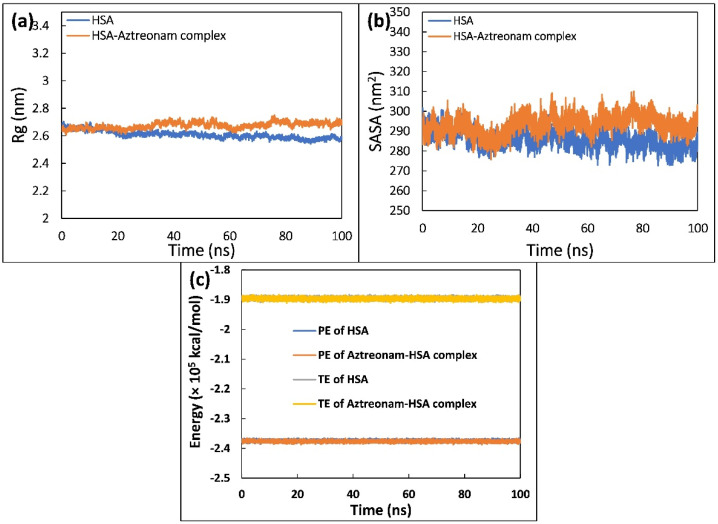
(**a**) Radius of gyration (Rg) of HSA and HSA-aztreonam complex as a function of time. (**b**) Solvent accessible surface area (SASA) of HSA and HSA-aztreonam complex as a function of time. (**c**) Potential energy (PE) and total energy (TE) of HSA and HSA-aztreonam complex as a function of time.

The radius of gyration (Rg) of uncomplexed and complexed HSA is shown in Figure 9a, which was calculated to examine the compactness of HSA in the free and ligand-bound state. HSA and HSA complexed with aztreonam were found to have average Rg values of 2.59 and 2.70 nm, respectively. Due to the occupancy of intramolecular space by aztreonam, the average values of Rg increased marginally, indicating that the structure of HSA seemed to have some minor loose packing. During the simulation, however, this slight increase does not appear to cause any significant conformational changes in the structure of HSA. Overall, the figure indicated that the HSA-aztreonam complex remained stable and consistent throughout the experiment [48].

Analyzing the change in solvent accessibility during the simulation was helpful in evaluating the folding dynamics [49]. Hence, SASA of HSA was calculated and plotted as a function of time during its interaction with aztreonam, as shown in Figure 9b. The plot clearly shows that following binding with aztreonam, SASA of HSA increases slightly, which could be attributed to the exposure of certain hidden residues. However, the folding of HSA during the simulation does not appear to be affected by this slight increase. Throughout the simulation trajectory, the SASA distribution exhibited a consistent equilibration with no major deviations.

Total and potential energies were also estimated to further validate the stability of the complex formation between HSA and aztreonam [50]. Figure 9c shows the potential energy (PE) and total energy (TE) of HSA and HSA-aztreonam complex as a function of time. The constant values of total energy and potential energy of HSA alone and HSA complexed with aztreonam show that the system remained stable during the simulation.

**Figure 10:**
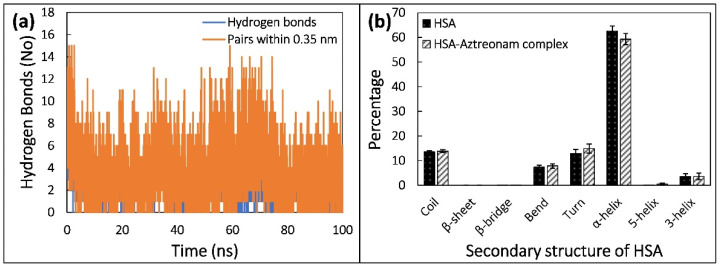
(**a**) Number of hydrogens formed by aztreonam with HSA as function of time. (**b**) Average secondary structural components of HSA in the absence and presence of aztreonam.

The stability of a protein structure with respect to conformation is determined by hydrogen bonds (H-bonds). As a result, the stability of HSA and HSA-drug complexes may be assessed using H-bonding studies. We explored the involvement of intra- and intermolecular H-bonds, as shown in Figure 10a, to further establish the stability of HSA and its complex with aztreonam during simulation. H-bond formation in HSA is stable, establishing protein shape even after aztreonam binding, according to the findings.

The MD trajectories were used to assess intermolecular H-bonding as well as the impact of aztreonam binding on the HSA structure. The formation of H-bonds between aztreonam and HSA was investigated and plotted over time. During the 100 ns simulation timeframe, aztreonam indicated the formation of many H-bonds with HSA.

Protein biological function is inextricably linked to secondary structure. To quantify the conformational changes, the secondary structure components were computed using the DSSP method. The a-helix proportions in free HSA are 62 percent, while in the HSA-aztreonam complex, they are 59 percent. This could indicate that aztreonam has the ability to bind with HSA polypeptide chain residues, partially disrupt hydrogen bonding networks, and cause protein instability [51]. Unfolding of native protein structure is indicated by a decrease in helicity and an increase in unordered coil regions. As a result of the interaction between aztreonam and HSA, the secondary structure composition changed. Figure 10b shows the % change in protein secondary structure after aztreonam and HSA interaction, which caused minor conformational and microenvironmental alterations in the protein. Overall, the data are comparable with the finding of CD analysis. The main difference was that no β-sheets were found in simulation data while roughly 10.9% and 21% β-sheets were obtained from CD data.

**Figure 11:**
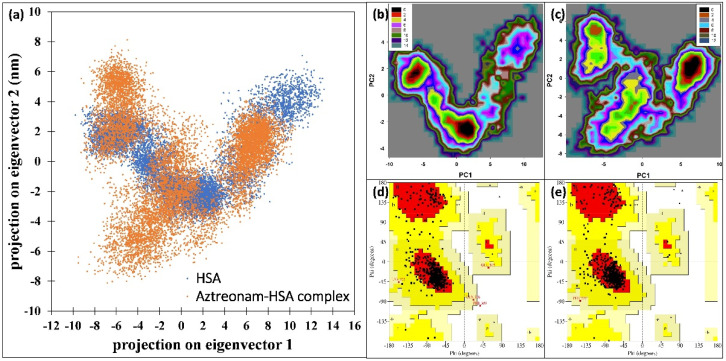
(**a**) 2D scatter plot of principal component analysis (PCA) by projecting the eigenvectors of HSA and HSA-aztreonam complex. (**b**) Free energy landscape plot of HSA. (**c**) Free energy landscape plot of HSA-aztreonam complex. (**d**) Ramachandran plot of HSA. (**e**) Ramachandran plot of HSA-aztreonam complex.

The impact of interaction of aztreonam on the collective motions in HSA was investigated using PCA using the first two eigenvectors (EVs). Figure 11a shows the scatter plots generated by the native HSA and HSA-aztreonam complexes. The conformational projections of HSA and HSA complexed with aztreonam showed no significant differences in the figure. When compared to the initial HSA, the projection of the HSA-aztreonam complex shrank on both EVs during the simulation. When resemblance to the native HSA, the HSA complexed with aztreonam explored a reduced phase space. Overall, the conformational motions in HSA alone and HSA-aztreonam systems were not varied, as indicated in the plot, implying complex stability [52].

The first two eigenvectors (PC1 and PC2) were used to perform a free energy landscape analysis to learn more about the protein conformational transition and least energy structure [53]. In the free energy landscape (FEL) plot (shown in black) in Figure 11b,c, there are two energy minima. From the energy minima, the lowest energy structures of the HSA-aztreonam complex were extracted. The Ramachandran plot of the energy minima structures were made, Figure 11d,e. There was only one amino acid occurring in disallowed region of HSA. However, no residue was found to be present in the disallowed region for HSA-aztreonam complex. The data further show the stable nature of the complex.

The binding free energy of aztreonam to HSA was calculated using the MM-PBSA method. The binding energy of MM-PBSA was calculated for every 5 ns MD simulation for 100 snapshots and is listed in Table 2. Different sorts of interactions arise between atoms of aztreonam and different residues of HSA based on the chemical nature of aztreonam. These interactions include hydrophobic, hydrogen, electrostatic, and polar interactions [54]. Each sort of interaction affects the overall binding energy (BE) in a favorable or unfavorable manner. Aztreonam has a strong affinity for HSA, having a binding affinity of −9.73 ± 0.48 kcal/mol, suggesting a stable complex formation between them.

The free energy of amino acid residues which play a key role in the binding process can be calculated from the aztreonam binding pose. The total binding energy and polar, apolar contributions of 9 key residues were calculated and the data are tabulated in Table 3. It shows that all the 9 residues work positively towards the total binding process.

## 3. Materials and Methodology

### 3.1. Material

Aztreonam (≥98%), HSA, ibuprofen (≥98%), and warfarin (98%) were obtained from Sigma-Aldrich (Bangalore, India). The HSA’s stock solution was prepared in sterile 10 mM sodium phosphate buffer (pH 7.4). The stock solution of ibuprofen (5 mM), warfarin (5 mM), and aztreonam (4 mM) were prepared in DMSO.

### 3.2. Methodology

#### 3.2.1. UV-Vis Absorption Spectroscopic Analysis

A double beam UV-vis spectrophotometer (Shimadzu UV1800, Kyoto, Japan), was used to measure all UV-vis spectra in 1 cm quartz cuvettes. A fixed concentration of has (5 µM) was titrated with aztreonam concentrations ranging from 0–50 µM and the absorbance was recorded at all concentrations. The absorption spectra were measured between 200 and 350 nm. All measurements were recorded against 10 mM sodium phosphate buffer as base line.

#### 3.2.2. Steady State Fluorescence Measurements

First, the fluorescence emission spectrum of HSA (5 µM) was recorded from 280 to 420 nm. A varying concentration of aztreonam (0–50 µM) was added and fluorescence emission signal was recorded at each addition. The spectrofluorometric measurements were taken on a Shimadzu RF-6000 spectrofluorometer (Kyoto, Japan). The excitation wavelength was fixed at 280 nm. All measurements were recorded at room temperature. The values of various quenching and binding parameters were calculated from the steady state fluorescence data.

#### 3.2.3. Site Marker Displacement Assays

Site marker displacement assays were done to decipher the site of binding of aztreonam in HSA. For site I, site II, and site III, warfarin, ibuprofen, and bilirubin were used, respectively. The increasing concentrations (0–30 µM) of each site marker were added to the complex of HSA (5 µM) and aztreonam (60 µM). Each site marker examination was carried out separately. The samples were excited at 280 nm and emission was monitored from 280 to 450 nm.

#### 3.2.4. 3D Fluorescence Spectroscopic Measurement

The 3D fluorescence spectra (Shimadzu RF-6000 spectrofluorometer, Kyoto, Japan) of HSA (5 µM) without and with the aztreonam (30 µM) were taken at room temperature. The emission signals were taken from 200 to 550 nm by exciting the samples from 200 to 400 nm.

#### 3.2.5. Circular Dichroism Spectroscopy

Using a JASCO (J-1500) CD spectrophotometer (Tokyo, Japan), the CD spectra of HSA (5 M) were obtained in the absence and presence of aztreonam. A quartz cuvette with a 0.1 cm path length was used and CD spectra was recorded ranging from 190 to 240 nm. Each spectrum was taken at 1 nm intervals and spectra were taken at room temperature. CD data were converted to molar residue ellipticity (MRE) for the calculation of % α-helix.

#### 3.2.6. FRET (Fluorescence Resonance Energy Transfer) Measurements

The fluorescence of HSA (30 µM) was taken from 200 to 600 nm and absorption spectra of aztreonam (30 µM) were recorded in same wavelength range. The data were normalized for further calculations. The energy transfer efficiency and distance were calculated using the spectral overlap data.

#### 3.2.7. Molecular Docking

The molecular docking of aztreonam with HSA was performed using AutoDock Vina [55]. The crystal structure of HSA was downloaded from RCSB (1AO6). The energy of protein was minimized using Swiss PDB viewer. The structure of aztreonam was obtained from PubChem (CID: 5459211). The crystal water was removed from HSA and then polar hydrogen atoms were added. The Kollman charges were also added to protein structure using MGLTools 1.5.6 (La Jolla, CA, USA) [56]. The structure of aztreonam was made flexible to obtain the best possible docking conformation. The size of grid was 74 × 60 × 82. The remaining docking parameters were left to their default values. The analysis of molecular docking results was done using Discovery Studio and PyMOL.

#### 3.2.8. Molecular Simulation

The molecular dynamic (MD) simulation of HSA alone and HSA-aztreonam complex was done using Gromacs-2018.1 and the force field amber99sb-ILDN was used. The topology of ligand was generated using antechamber package of AmberTools18 [57]. Both structures (uncomplexed and complexed structures) were solvated in triclinic boxes using TIP3P water model. The counter ions were used to neutralize the system and then 150 mM NaCl was added. Then, 100 ns MD simulation was completed and trajectories were stored at equal intervals. Various binding energies were calculated using MM-PBSA analysis [58].

### 3.3. Statistical Analysis

All in vitro experiments were performed in three independent replicates. The data are provided as the average with standard deviation.

## 4. Conclusions

Various spectroscopic and computational techniques were used to reveal a thorough interaction mechanism of aztreonam with HSA and its effect on conformational as well as structural stability of HSA in this paper. The formation of a complex between HSA and aztreonam was established by UV-vis absorption and fluorescence data. The binding constant was determined to be in 10^4^ M^−1^ range, which is moderate in strength. Aztreonam binds at site III of has, according to site marker displacement investigations. Molecular docking analyses supported these findings even further. The distance between the HSA and aztreonam was optimal for energy transfer as evident from FRET data. The results of 3D fluorescence and CD measurements revealed changes in microenvironment and the secondary structure of HSA due to aztreonam binding. Furthermore, molecular simulation results depicted that hydrogen bond, van der Waal interaction, and pi–anion interaction are the forces associated with the process of binding and in the stability of complex formation.

## Figures and Tables

**Table 1:** Data derived from three-dimensional excitation-emission matrix fluorescence of HSA and HSA-aztreonam system.

Peak Name	Peak Position (nm) (λ_ex_ = λ_em_)	Stokes Shift (Δλ)	HSA Alone	HSA:Aztreonam (1:6)	F/F_0_
**Peak ‘a’**	280/280	---------	901,452.3	410,424.5	0.455
**Peak ‘b’**	280/540	---------	34,550.4	8556.4	0.248
**Peak ‘1′**	280/340	60	135,692.1	119,887.8	0.884
**Peak ‘2′**	225/345	120	72,526.4	37,189.7	0.513

**Table 2:** Binding free energy (kcal mol^−1^) for the interaction of aztreonam with HSA using MMBSA analysis.

Type of Energy	Energy
**ΔE_vdW_**	−31.69 ± 0.56
**ΔE_ele_**	−18.58 ± 0.82
**ΔE_PSE_**	44.40 ± 1.18
**ΔES_SASA_**	−3.86 ± 0.05
**ΔE_BE_**	−9.73 ± 0.48

ΔE_vdW_: van der Waal energy, ΔE_ele_: electrostatic energy, ΔE_PSE_: polar solvation energy, ΔE_SASA_: solvent accessible surface area energy, ΔE_BE_: binding energy.

**Table 3:** The average polar, apolar, and total binding energies (kcal mol^−1^) of the key residues.

Residues	E_polar_	E_Apolar_	E_total_
Arg-117	0.15 ± 0.24	−0.13 ± 0.01	−1.17 ± 0.14
Pro-118	0.13 ± 0.04	−0.16 ± 0.00	−1.12 ± 0.07
Arg-145	0.98 ± 0.28	−0.03 ± 0.00	−0.44 ± 0.08
Leu-179	1.63 ± 0.05	−0.20 ± 0.00	−1.14 ± 0.10
Pro-180	0.31 ± 0.01	−0.07 ± 0.00	−0.55 ± 0.05
Leu-182	0.00 ± 0.02	−0.06 ± 0.00	−0.81 ± 0.02
Arg-186	1.47 ± 0.22	−0.34 ± 0.00	−2.75 ± 0.18
Lys-190	0.59 ± 0.19	−0.01 ± 0.00	−0.46 ± 0.08
Ile-523	0.00 ± 0.00	−0.06 ± 0.00	−0.44 ± 0.04

E_polar_: polar energy; E_Apolar_: apolar energy; E_total_: total energy.

## Data Availability

Not applicable.
